# Isolation and Characterization of 15 New Microsatellite Markers in *Oncomelania hupensis*, the Snail Intermediate Host of *Schistosoma japonicum* in Mainland China

**DOI:** 10.3390/ijms13055844

**Published:** 2012-05-15

**Authors:** Li Zhang, Shizhu Li, Qiang Wang, Yingjun Qian, Qin Liu, Pin Yang, Xiaonong Zhou

**Affiliations:** National Institute of Parasitic Diseases, Chinese Center for Disease Control and Prevention, Shanghai 200025, China; E-Mails: zhangli5337@gmail.com (L.Z.); qiangwang79@163.com (Q.W.); yjqiancn@gmail.com (Y.Q.); liuqin0901@sohu.com (Q.L.); pinyang1980@hotmail.com (P.Y.); xiaonongzhou1962@gmail.com (X.Z.)

**Keywords:** *Oncomelania hupensis*, microsatellites DNA, polymorphism, population genetics

## Abstract

*Oncomelania hupensis* is the unique intermediate host of *Schistosoma japonicum*, which plays a key role during the transmission of schistosomiasis. It is mainly found in the Yangtze River valley and mountains or hills in southwest China. In this paper, we described 15 new microsatellite makers in *O. hupensis.* Polymorphism of each locus was assessed in 80 individuals from four wild populations (*n* = 20 per population). The number of alleles per locus ranged from 6 to 29, with an average of 15.8. The observed (*H_O_*) and expected (*H_E_*) heterozygosities varied from 0.397 to 0.851 and from 0.696 to 0.948, respectively. These microsatellite markers will be useful for population genetic studies and genome mapping in *O. hupensis*.

## 1. Introdution

Schistosomiasis, caused by *Schistosoma japonicum*, remains one of the most prevalent parasitic infections and raises significant socio-economic and public health consequences in China [1–4]. *Oncomelania hupensis* is the unique intermediate host of *Schistosoma japonicum*, which plays a key role during the transmission of schistosomiasis and mainly spread in the Yangtze River valley and mountains or hills in southwest China [5–7]. The *O. hupensis* has been found in four different ecological settings: the region of swamps and lakes in the Yangtze River basin (part of the Anhui, Hubei, Hunan, Jiangsu, Jiangxi and Zhejiang provinces), the mountainous region of the Sichuan and Yunnan provinces, the hilly, littoral part of the Fujian province, and the Karst landscape of Guangxi autonomous region. *O. hupensis* has caused a schistosomiasis endemic in mainland China [8,9]. The genetic diversity in the different geographical populations of the snail and the co-evolution between *O. hupensis* and *S. japonicum* are of great interest because the branching patterns of snail diversity could be a map to the patterns of parasite diversity [10–12]. However, microsatellite markers have not been used extensively for the snail genetic structure studies and genome mapping. There are only a few applications of SSR-PCR analysis of genetic variation in different populations [13,14].

## 2. Results and Discussion

A total of 292 positive clones were identified and sequenced. Out of the 292 sequences, 26 sequences did not contain microsatellite sequences. Sixty-one sequences were not used because the length was less than 100 bp or they were the same sequences. Therefore, in total, 205 unique sequences were obtained (GU204044- GU204248). From 30 chosen sequences, a total of 21 primer pairs produced successful and consistent amplification. These primers were further examined for polymorphism with *O. hupensis* from Fujian (FJF), Sihuan (SCH), Guangxi (GXY), and Anhui (AXH) populations, and 20 individuals were taken from each population. Fifteen microsatellite loci displayed polymorphisms (Table 1).

The number of alleles per locus ranged from 6 to 29, with an average of 15.8. However, three loci (T5-11, D11, T4-36) were monomorphic in the GXY population. The observed (*H_O_*) and expected (*H_E_*) heterozygosities varied from 0.397 to 0.851 and from 0.696 to 0.946, respectively. Significant deviation from Hardy–Weinberg equilibrium (HWE) was observed, 13 out of 60 (21.67%) possible single exact locus tests (*P* < 0.01). Analysis with MICROCHECKER indicated the possible occurrence of null alleles at six loci (T6-47, T5-21, T4-36, E3, E15, C23). The presence of null alleles can sometimes be detected as an excess of homozygotes leading to deviations from HWE. In addition, null alleles lower apparent genetic variability, they may erroneously inflate levels of genetic differentiation and affect population genetic analyses that rely on HWE [15,16]. A deviation from HWE may also be due to selection, population mixing, nonrandom mating, sampling strategies, and undetected sex-linkage. No significant linkage disequilibrium was found between all pairs of these 15 loci (Table 2) (*P* < 0.01), which indicated the independent behavior of all loci.

## 3. Experimental Section

### 3.1. Isolation of Microsatellite Loci

All the samples were bred in a laboratory for at least one week. Then, the samples negative to *S. japonicum* were selected. After removal of the gut and digestive glands from the soft parts of the snail, genomic DNA was extracted from the muscle tissues of the snail followed by the standard DNA extraction procedure using mollusk DNA Kit (Omega, Norcross, GA, USA) [17]. Then, we followed the protocol of Hammond for construction of a microsatellite enriched genomic library, with some minor modification [18]. Briefly, total genomic DNA was digested with restriction enzyme Sau3AI (Fermentas, Burlington, Ontario, Canada) and then ligated to Sau3AI AFLP adaptor followed by amplification with adaptor-specific primers (*Sau*LA: 5′-GCG CTA CCC GGG AAG CTT GG-3′, and *Sau*LB: 5′-ATC CCA AGC TTC CCG GGT ACC GC-3′).

Microsatellite enrichment involved three rounds of PCR amplification. The first enrichment PCR used *Sau*LA sequence as primer and ligated DNA as template. The amplified DNA fragments were then denatured and hybridized with biotinylated oligonucleotides [(AAT)17, (GA)25, (CCT)17, (AC)25, (CAG)17, (CAC)5, (TC)10, (TG)18]. The target moleculars bond with complementary microsatellites in the genomic library. The genomic fragments with microsatellite were captured with Vectrex Avidin D through hybridization. Genomic DNA that contained microsatellite repeats were stripped from Vectrex Avidin D and concentrated by ultrafiltration using Amicon Ultra-4 (Millipore). The second enrichment was identical to the first enrichment procedure to further amplify genomic fragments with microsatellites. The third enrichment procedure contained PCR amplification only. The PCR used the SauLA sequence as primer and concentrated DNA from the second enrichment as template. The amplified fragments were cloned into a plasmid using TOPO TA Cloning Kit (Invitrogen), and a microsatellite-enriched genomic library was thus constructed.

### 3.2. Detection of Polymorphism

Thirty sequences were chosen and used to amplification. Primers were designed flanking each suitable microsatellite sequence, using the primer 3.0 computer program [19]. A total of 21 primer pairs produced successful and consistent amplification, and those primers were further examined for polymorphism with *O. hupensis* field snails. One of the two primers that were used to amplify each locus was labeled with a fluorescent dye such as HEX, NED and FAM. Polymorphisms of microsatellite loci were evaluated in 80 wild individuals *O. hupensis* from Fujian, Sichuan, Guangxi and Yunnan province. Microsatellites were amplified under the following conditions. The reaction mixtures (25 μL) total containing 1× Taq buffer, 0.15 mM dNTPs, 0.5 μM forward and reverse primers, 1.5 mM MgCl_2_, 1.0 U Taq polymerase (Tiangen, Beijing, China) and about 20 ng gemonic DNA. PCR amplification was carried out on a Thermal Cycler (PTC-100, BIO-RAD, USA). Conditions included the following steps: an initial denaturation at 95 °C for 5 min, 35 cycles of 95 °C for 1 min, annealing at a set temperature depending on each locus (Table 1) for 45 s and 72 °C for 1.5 min, and final extension at 72 °C for 5 min. PCR products were determined using the Genetic Analyser 3730 (Applied Biosystems, Carlsbad, CA, USA), and analyzed by genescan 3.7 and genotyper 3.7 (Applied Biosystems).

### 3.3. Data Analysis

Standard genetic diversity parameters of polymorphic loci, e.g., the number of alleles (*N_A_*), and expected (*H_E_*) and observed (*H_O_*) heterozygosity, and Hardy-Weinberg equilibrium (HWE) and linkage dis-equilibrium were tested using GENEPOP 4.0 [20]. Null allele frequencies were calculated using Micro-Checker 2.2.3 [21].

## 4. Conclusions

The 15 microsatellite markers developed in this study are the first set of such markers for *O. hupensis*. They should prove useful for further investigating the spatial genetic structure, genetic diversity, and levels of gene flow within and among populations of this species.

## Figures and Tables

**Table 1:** Characteristics of the 15 microsatellite loci in *Oncomelania hupensis*.

Locus	GenBank Accession No.	Primer Sequence (5′→3′)	Repeat Motif	Ta (°C)	Allele Size from Field Snails (bp)
P82	GU204045	Pf: AAGAACTGCTCATACTGGAAAG Pr: GTGGTGCCCCTACGACCT	(GGA)_4_(GAA)_12_	51	176–242
T4-22	GU204083	Pf: TATCCAAGAAGCCGAAAC Pr: GAGGAAAGCGAGGTAAGA	(CA)_10_CC(CA)4	50	224–256
T5-11	GU204092	Pf: ACGCCAGTCTTGGTGTCA Pr: TACTTGGGCAGAAGGGTT	(TG)_14_TA(TG)_4_	55	137–165
D11	GU204223	Pf: AGCTTGGGATCAGAATGTCGTTTGT Pr: TATGTAGATGTTCACTGGTTTGTCC	(TG)_17_	55	172–192
T6-27	GU204213	Pf: AATGACACCCCGAACAAA Pr: CACTTCTCAACTCCAACCT	(TG)_6_G(GT)_6_..(GT)_12_	55	178–210
T6-17	GU204108	Pf: GGCCTGCCTTGGTTTTTTCACGTAG Pr: AGCTTGGGATCATCTCCAGGTC	(AC)_8_	55	230–248
B14	GU204050	Pf: CAGTCACAGCGCAGCCTACGA Pr: TCAAGCGACCTGATGTCAAATACC	(AG)_33_	55	151–259
T4-33	GU204086	Pf: GTCAAAACAACGAGGGCTGT Pr: CTGAGTGGAATGGGAGTTGG	(AC)_19_	60	135–173
C22	GU204145	Pf: TGGGATCGGTACATCTGGATAGTGG Pr: GGGATCAATGAAAGTTCTTGCGTTC	(CA)_21_	62	210–263
T6-47	GU204215	Pf: CCGAAGTGATAGAAACCG Pr: AGGCAGAAATGGGCAGAC	(TG)_7_...(GT)_9_	55	172–202
T5-21	GU204196	Pf: ATAAGTTTAGCCAGTCACCC Pr: ACACGCAGTCCACGCACA	(GT)_16_GG(GT)_4_T T(GT)_7_TT(GT)_4_	55	155–185
E3	GU204069	Pf: GATTTGTGAAAGTGAGGGTA Pr: TAGCAGGCGTCAAGGTAA	(CA)_33_	55	201–229
E15	GU204173	Pf: AAAGAACCGAATCAGGAC Pr: TACCAGCCGATGAATAAA	(AC)_22_CC(AC)_13_ AT(AC)_8_	55	123–225
C23	GU204058	Pf: CTGGACCTAAAGCAATAAC Pr: GAGCCAATCACCTAAACTA	(GT)_14_	55	144–188
T4-36	GU204088	Pf: CGGGTTACGGGAAAGGAT Pr: AGGGACGAACTCACGAAG	(CA)_17_	55	192–250

**Table 2:** Parameters of genetic diversity of *Oncomelania hupensis* at different loci.

Population	Index	Microatellite Locus															Total

		P82	T4-22	T5-11	T6-27	T6-17	D11	B14	T4-33	C22	T6-47	T5-21	T4-36	E3	E15	C23	
FJF	*Na*	5	6	6	7	8	3	11	5	4	8	5	5	7	4	8	6.13
	*H_O_*	0.563	0.733	1	0.563	0.75	0.75	0.813	0.882	0.722	0.611 *	0.667	0.5	0.611	0.722	0.778 *	0.727
	*H*_E_	0.752	0.779	0.845	0.802	0.821	0.605	0.895	0.791	0.751	0.889	0.765	0.818	0.737	0.751	0.8	0.711

SCH	*Na*	9	4	10	5	10	5	12	5	6	9	4	10	4	6	4	6.87
	*H_O_*	0.667	0.556	0.889	0.75	0.889	0.684	0.722	0.842	0.944	0.722 *	0.563	0.611 *	0.556	0.5 *	0.444	0.689
	*H*_E_	0.833	0.684	0.886	0.792	0.864	0.721	0.914	0.794	0.795	0.886	0.77	0.883	0.706	0.802	0.751	0.805

GXY	*Na*	8	8		6	6	1	10	3	5	7	7	1	7	4	3	5.13
	*H_O_*	0.526	0.737	1	0.632	0.895	0	0.737	1 *	0.833	0.556 *	0.5 *	0	0.333 *	0.444	0.4	0.506
	*H*_E_	0.743	0.741	0	0.828	0.657	0	0.862	0.539	0.697	0.846	0.822	0	0.718	0.614	0.481	0.57

AHX	*Na*	8	0	6	11	9	4	8	8	8	9	8	6	4	9	8	7.06
	*H_O_*	0.722	0.882 *	0.526	0.778	0.684	0.526	0.889	0.684	0.684	0.737 *	0.611 *	0.474	0.444	0.579	0.5 *	0.624
	*H*_E_	0.776	0.758	0.797	0.895	0.859	0.627	0.819	0.841	0.788	0.838	0.854	0.812	0.687	0.865	0.806	0.801

Total	*Na*	18	13	13	23	19	6	29	13	16	16	15	18	10	14	14	15.80
	*H_O_*	0.62	0.725	0.583	0.681	0.806	0.479	0.789	0.851	0.794	0.658	0.586	0.397	0.486	0.562	0.536	0.637
	*H*_E_	0.893	0.796	0.871	0.948	0.907	0.696	0.939	0.893	0.9	0.903	0.902	0.895	0.739	0.864	0.873	0.868

*N_A_*, number of alleles; *H_O_*, observed heterozygosity; *H_E_*, expected heterozygosity;

* Statistically significant deviation from Hardy–Weinberg equilibrium (*P* < 0.01).
